# Floral scent of artificial hybrids between two *Schiedea* species that share a moth pollinator

**DOI:** 10.1002/ajb2.70065

**Published:** 2025-06-29

**Authors:** John M. Powers, Stephen G. Weller, Ann K. Sakai, Diane R. Campbell

**Affiliations:** ^1^ Department of Ecology and Evolutionary Biology University of California at Irvine 321 Steinhaus Hall, Irvine California 92697 USA

**Keywords:** Caryophyllaceae, chemical ecology, Erebidae, floral volatile organic compounds, Hawaiian Lepidoptera, hybridization, oceanic island pollination, pollinator specificity, postzygotic barriers, reciprocal crosses

## Abstract

**Premise:**

In flowering plants, pollinators' ability to recognize chemical displays of hybrids may erode reproductive barriers. Hybrids may produce novel or altered floral scent blends that are unattractive, or scents similar to either parent that remain attractive and promote backcrossing. We characterized the floral scent of hybrids of sympatric species with a shared pollinator and tested whether scent is sufficient for pollinator attraction.

**Methods:**

Floral volatiles of artificial F_1_ hybrids between Hawaiian *Schiedea kaalae* and *S. hookeri* (Caryophyllaceae) were characterized by dynamic headspace sampling and GC‐MS. Behavioral choice tests with the native moth *Pseudoschrankia brevipalpis* measured the effect of adding *S. kaalae* scent (with flowers bagged to remove visual cues) to inflorescences of relatively unattractive wind‐pollinated relatives (*S. kealiae* and *S. globosa*) from the same island.

**Results:**

Most hybrids produced a combination of the distinct sets of floral volatiles from each parent at rates of emission that often differed from the expectation under completely additive inheritance. Floral scent did not depend on cross direction, and no novel compounds were detected in hybrids. *Pseudoschrankia brevipalpis* preferred inflorescences of *S. globosa* and *S. kealiae* that were augmented with the scent of hidden *S. kaalae* flowers.

**Conclusions:**

Intermediate hybrid floral scent blends could potentially attract moths if they do not rely on precise compound ratios. Moth attraction to the floral scent of *S. kaalae* flowers indicates that moths can discriminate the floral scent of this species against a background of volatiles and visual cues from wind‐pollinated relatives, showing the importance of scent variation in this genus.

Several sequential reproductive barriers maintain the genetic boundaries between plant species, and some act after hybrids are formed by affecting their ability to reproduce. When related species lack strong prezygotic barriers (including distinct habitats, phenology, pollinators, and post‐pollination prezygotic isolation), these late‐acting postzygotic barriers can still prevent gene flow (Rieseberg and Willis, 2007; Wendt et al., 2008; Widmer et al., 2009). Conversely, the ability of hybrids to reproduce with other hybrids or parent species would break down species barriers and reverse genetic differentiation among taxa.

Pollinator specificity is a key component of reproductive isolation often mediated by species‐specific floral displays. Displays advertise rewards to pollinators and include the release of informative scents that are especially important to attract nocturnal pollinators (Miyake et al., 1998; Borges et al., 2016). The floral scent produced by hybrids in comparison to their parent species could affect their pollination, especially if the parental scents are distinct from each other (García et al., 2023). On one extreme, novel hybrid scents that are unattractive to pollinators of the parents but attractive to new pollinators can lead to immediate reproductive isolation with parents, as shown in natural hybrids of sexually deceptive orchids (*Orphys*, Vereecken et al., 2010) and food‐rewarding daffodils (*Narcissus* [Amaryllidaceae], Marques et al., 2016). Alternatively, hybrid scent that still attracts parental pollinators could enhance backcrossing with one or both parents, leading to introgression, as shown in a Joshua tree contact zone (*Yucca* [Asparagaceae], Svensson et al., 2016).

The attractiveness of hybrid scent blends depends on their chemical composition, which could be influenced by the heritability of volatile production (Zu et al., 2015), whether inheritance is additive or shows allelic dominance or epistasis (Byers et al., 2014), and mechanisms of regulation. Hybrid individuals can produce volatile blends similar to one parent (Svensson et al., 2016; Zito et al., 2018), quantitatively intermediate blends (Campbell et al., 2016; Svensson et al., 2016), transgressive blends outside the phenotypic range of both parents (Bischoff et al., 2014; Marques et al., 2016; Rubini Pisano et al., 2018), novel volatile compounds (Vereecken et al. 2010; Marques et al., 2016), or reduced total scent emissions (Hirota et al., 2012). The attractive abilities of these scent blends relative to parental species depend on pollinator olfaction and preferences, with some pollinators requiring compounds mixed in precise ratios and others only requiring the presence of key compounds (Wright et al., 2005).

Many previous studies examined scents in natural hybrid populations, which may have already experienced selection for floral traits preferred by pollinators, and so could lead to an overestimate of the attractiveness of hybrids when they are first formed. Natural hybrid populations also include advanced‐generation hybrids and backcrosses in heterogeneous environments that could affect scent expression, so the link between novel genetic combinations and hybrid floral phenotypes is less clear (Bischoff et al., 2014; García et al., 2023; Albuquerque‐Lima et al. 2024). These problems are avoided through study of artificial F_1_ hybrids resulting from crosses that are raised from seed under controlled conditions (Fu et al., 2007; Hirota et al., 2012; Okuyama, 2015; Wei et al., 2021; García et al., 2023). Because hybrids arising from a particular cross direction may be expected at sites of each parent species, this approach also allows comparisons of reciprocal crosses (Wei et al., 2021).

Little is known about the role of scent versus visual attraction in flowers pollinated by small moths, or those that lack the conspicuous white petals typical of the nocturnal moth pollination syndrome. Insects often integrate both olfactory and visual cues to locate flowers (Chittka and Raine, 2006; Barragán‐Fonseca et al., 2020), and large hawkmoths use combinations of these cues for upwind flight and close‐range location of flowers (Raguso and Willis, 2002, 2005; Raguso, 2008; Riffell and Alarcón, 2013). However, bioassays with synthetic scents show that scent alone is sufficient to attract some pollinators (Knudsen et al., 1999; Peakall et al., 2010), and small moths may rely on different sensory modalities than hawkmoths. For example, a study of pollinator attraction to the small, greenish‐white flowers of *Silene otites* (L.) Wibel (Caryophyllaceae) in the absence of visual cues showed that floral scent alone attracted small moths and other insects (Dötterl et al., 2012). The endemic lepidoptera of Hawaiʻi are particularly diverse with over 900 species described (Zimmerman, 1958; Ziegler, 2002) and known or inferred to be critical for pollinating endangered plant populations (Norman et al., 1997; Newbery et al., 1998; Elmore, 2008; Weisenberger et al., 2014; Medeiros, 2015; Weller et al., 2017; Shay and Drake, 2018; Aslan et al., 2019; Walsh et al., 2019), yet the mechanism of their attraction to flowers has not been studied.

In this study, we evaluated the floral scent of hybrids of two sympatric Hawaiian plant species, *Schiedea kaalae* Wawra and *S. hookeri* A. Gray (Caryophyllaceae). For these species, our knowledge of both insect pollination and the composition of floral volatile production exceeds that of any other species in the Hawaiian flora, yet the mechanism of pollinator attraction and the effects of hybridization in shaping floral scent remain unknown. These *Schiedea* species share a small endemic moth pollinator, which is the only known pollinator of *S. kaalae*. The two species lack petals or other showy ornamentation (Appendix S1) and produce unique floral scents that intensify and diverge from each other in the evening (Weisenberger et al., 2014; Weller et al., 2017; Powers et al., 2020). We determined whether the evening scent emissions of bidirectional artificial hybrids are identical to one parent, outside the range of either parent, intermediate, reduced, increased, or include novel compounds.

While due to conservation concerns, we could not test pollinator preferences for hybrids against their parent species in the field (as in Wesselingh and Arnold, 2000; Campbell et al., 2002; Ippolito et al., 2004), we verified that floral scent influences moth behavior. We used field choice experiments to establish that the evening floral scent of *S. kaalae* in the absence of visual cues is sufficient to attract moths against a background of visual and olfactory cues from either of two wind‐pollinated *Schiedea* species that are relatively unattractive to moths (Weller et al., 2017). If species‐specific olfactory cues can attract pollinators, then altered floral scent production in hybrids may contribute to maintaining the boundary between these species.

## MATERIALS AND METHODS

### Hybridization in *Schiedea*

*Schiedea* is a monophyletic genus that has radiated across the Hawaiian Islands and includes 34 extant species with diverse pollen vectors (moths, wind, and presumably birds) and breeding systems (e.g., hermaphroditism, dioecy, autogamy; Wagner et al., 2005; Sakai et al., 2006). Early‐acting postzygotic viability barriers are generally weak in the genus, i.e., all tested crosses between 17 species produced seeds and vigorous F_1_ progeny (Weller et al., 2001). Some *Schiedea* species may produce hybrid zones, i.e., out of ten instances of current sympatry (Wagner et al., 2005), five instances of extant natural hybridization have been observed based on morphology (Weller et al., 2001), and seven instances of current and historical introgression between *Schiedea* species have been detected through molecular methods (two of the seven were already known from morphological intermediates, Soltis et al., 1996; Wallace et al., 2011; Willyard et al., 2011).

### Focal species

*Schiedea kaalae* (sect. *Mononeura*) and *S. hookeri* (sect. *Schiedea*) are hermaphroditic, self‐compatible, protandrous, perennial herbs or subshrubs (respectively) native to Oʻahu, Hawaiʻi, USA, where populations of the two species occur in sympatry in parts of the Waiʻanae Mountains (*S. kaalae* [410–730 m a.s.l.] and *S. hookeri* [260–870 m a.s.l.]; Wagner et al., 2005) and can flower at the same time. *Schiedea kaalae* also occurs in the Koʻolau Mountains, and a single collection of *S. hookeri* is known from West Maui (Wagner et al., 2005). These species occur in different clades that diverged c. 1.3 Mya based on a root age of 7.3 Mya for the genus (Willyard et al., 2011). Both species are listed as endangered by the US Fish and Wildlife Service and critically endangered by the IUCN (Ellshoff et al., 1991; Bruegmann and Caraway, 2003; Wagner et al., 2005; Bruegmann et al., 2016), and a total of only about 28 *S. kaalae* individuals in five populations remained in the wild before restoration efforts (Weisenberger et al. 2014). *Schiedea hookeri* is more common in nature than *S. kaalae*, and large populations also exist following restoration efforts (Dan Sailer, Oʻahu Army Natural Resources Program, personal communication). Both species possess similar floral morphology with reflexed sepals 3–4 mm long, no petals, 10 stamens, 3 styles, and 5 nectaries (Wagner et al., 2005; Appendix S1).

When artificially crossed, F_1_ hybrid seeds between sympatric *Schiedea kaalae* and *S. hookeri* germinate and flower in greenhouse conditions. Hybrids produce an intermediate number of inflorescences of intermediate biomass, and their total flower number is similar to *S. hookeri* (unpublished data). Flowers visually resemble both parents in shape and size (Appendix S1). Despite overlap in geographic range and phenology, and sharing the same pollinator species (Wagner et al., 2005; Weller et al., 2017, see below), no evidence of natural hybridization or past genetic introgression between these taxa has been detected (Wagner et al., 2005; Willyard et al., 2011). Absence of hybrids might result from the current rarity of *S. kaalae* or the existence of postzygotic barriers affecting hybrid reproduction.

### *Schiedea* pollination and floral scent

Pollination by small native moths has been observed in two species of *Schiedea* (Weller et al., 2017). Most *Schiedea* species have specialized tubular nectary extensions (Harris et al., 2012; Appendix S1) that are probed by moths to remove nectar (Weller et al., 2017). The two focal species of this current study, *Schiedea kaalae* and *S. hookeri*, are pollinated by the moth *Pseudoschrankia brevipalpis* Medeiros (Erebidae; Weller et al., 2017). The elliptic flight patterns of these moths before they land on flowers suggest they rely little on visual targeting even before dark and are characteristic of moths seeking floral volatiles through anemotaxis (upwind flight; Cardé and Willis, 2008; Weller et al., 2017 and videos therein). When beginning their foraging activity in the evening, moths fly upwind as they approach the experimental populations and can navigate to inflorescences that are out of sight (A. Sakai, personal observations). Despite sharing a primary pollinator, *S. kaalae* and *S. hookeri* produce distinct scent blends from each other, dominated by different compound classes, but also share some volatiles in common (Powers et al., 2020). Many volatiles produced by both species show a peak evening output that corresponds to the time of peak pollinator activity, and include common insect attractants such as indole, phenylacetaldehyde, and linalool derivatives (Powers et al., 2020). At a site planted exclusively with *S. kaalae*, *P. brevipalpis* preferred *S. kaalae* inflorescences (69% of inflorescence visits in choice tests, Weller et al., 2017) over *S. hookeri* inflorescences, demonstrating that moths can discriminate between the species. That study did not identify whether olfactory or visual cues are used. Moreover, this moth preference may only be a local, learned preference as the reciprocal test in a *S. hookeri* population has not been performed.

In addition to discriminating between *S. kaalae* and *S. hookeri*, *P. brevipalpis* prefers to visit either of those species that develop relatively large nectaries over two related wind‐pollinated species that occur on the same island (Oʻahu; *S. kealiae* Caum & Hosaka and *S. globosa* H.Mann, Weller et al., 2017). The two wind‐pollinated species have small largely vestigial nectaries and produce scents that are distinct from the scents of the two moth‐pollinated species (Jürgens et al., 2012; Powers et al., 2020, 2022).

### Floral scent of hybrids

#### Crossing design

Plants of the two species were grown in the greenhouse from seeds or cuttings of six populations from the Waiʻanae Mountains (10 *S. kaalae* and 10 *S. hookeri* plants, Appendix S2; all collections were made before the species were listed as federally endangered in 1991 and 1996, for *S. kaalae* and *S. hookeri*, respectively). Plants also were grown from intraspecific and interspecific (hybrid) crosses between cultivated plants from these populations (see Appendix S3 for sample sizes and Appendix S1 for photos of the flowers). To produce the plants of *S. kaalae* and *S. hookeri*, we used intrapopulation and interpopulation crosses within species because most natural populations now consist of a single individual and are highly inbred (Weisenberger et al., 2014). We conducted crosses between populations in both directions (e.g., with a plant from one species acting as the maternal parent and the plant from the other species acting as the paternal parent and vice versa) to examine potential nuclear‐cytoplasmic interactions or the direct effects of the two species’ cytoplasmic genomes on floral scent (hereafter, reciprocal effects), which has rarely been studied (Campbell et al., 2022). Since maternal environmental effects were presumably eliminated or greatly reduced by the cultivation history, in the absence of these cytoplasmic effects or interactions, scent composition and total emission rates would be similar for both cross directions. The hybrid crosses are denoted with the initial of the maternal parent first, e.g., H × K for a hybrid with *S. hookeri* as the maternal parent and *S. kaalae* as the paternal parent.

#### Volatile sampling

Floral volatile emissions of F_1_ hybrids were measured in the evening using the protocols for dynamic headspace collection, GC‐MS, and data processing methods used in Powers et al. (2020) and summarized here (sample sizes in Appendix S3). That previous study focused on temporal changes in floral scent and analyzed only the parental species, not the hybrids as we do here. Scent samples were collected from November 2016 to April 2017 in the greenhouse to provide a common environment for observing genetic differences. Because scent composition and amount depend strongly on time of day in both species (Powers et al., 2020), only samples taken 0–4.5 hr after sunset (mean ± standard deviation 2.0 ± 0.9 hr after sunset, corresponding to 17:15 to 21:45 local time) were used. This corresponds to the timing of moth activity in both species (Powers et al., 2020). Dynamic headspace samples of floral volatiles were taken by enclosing inflorescences in oven bags, allowing volatiles to equilibrate for 30 min at 20–26°C and pumping for 30 min through a Tenax scent trap with a pre‐trap flow rate of 200 mL/min. The numbers of open male‐ and female‐phase flowers closed (post‐anthesis) flowers, and floral buds were recorded immediately after sampling. All plants chosen had ≥ 10 open flowers. To assess the magnitude of within‐plant variation, for 13 plants multiple samples were taken from different inflorescences on different dates (29 inflorescences total). Because these resamples yielded similar scent emissions (Appendix S4), they were averaged within a plant before statistical analysis. Ambient controls (n = 19) were taken from an empty oven bag sampled for the same duration to identify contaminants (see below). Floral scent emissions were quantified by thermal desorption gas chromatography‐mass spectrometry (TD‐GC‐MS; see Powers et al., 2020 for details). Volatile emission rates were calculated within each compound class from peak integrations by calibration with dilutions of authentic standards. We analyzed the same filtered set of compounds as in Powers et al. (2020), plus (Z)‐hex‐3‐en‐1‐ol which was previously excluded as a potential wound volatile but differs in emissions between the species. The filters required compounds to occur in two or more samples, exceed a minimum peak area threshold in at least one sample, and have mean emissions in floral samples greater than ambient controls (as determined by t‐tests using a false discovery rate correction). Emission rates were standardized by the number of open flowers.

#### Statistical analysis of floral scent

To determine how scent intensity in hybrids related to the parent species, we compared emissions rates of each volatile and total emissions for the two species and two cross directions. If genetic control of floral volatiles emissions is additive, meaning there are no effects of allelic dominance or epistasis, then the mean emissions of hybrids (mean of both cross directions) will equal the mean of the two parental species. We tested for deviations from this additive expectation using a generalized linear hypothesis test in the *multcomp* R package, using the false discovery rate method for comparisons of each volatile (Hothorn et al., 2008). We also tested for a difference between the hybrid cross directions that would indicate reciprocal effects. We applied both tests to analyze volatile diversity, focusing on the number of volatiles (richness), and Shannon diversity of the blend (which captures both richness and evenness).

To analyze multivariate patterns of scent composition among plants, we performed a non‐metric multidimensional scaling (NMDS) with Bray‐Curtis distances between relative volatile emission rates, after applying a square‐root transformation (*vegan* R package, Oksanen et al., 2024). To test whether the hybrid cross directions differed in relative scent composition, a PERMANOVA was conducted between the two cross directions using the *adonis* function of *vegan*. To assess whether levels of beta diversity (variation in relative scent compositions among plants) differed among the two parent species and hybrids, we conducted an analysis of multivariate homogeneity of group dispersions using the *betadisper* function of *vegan* with Bray‐Curtis distances. Among‐plant Bray‐Curtis distances were also compared to within‐plant (among‐inflorescence) distances observed in the resampled plants.

To compare emission rates of individual volatiles between the groups in more detail and identify which volatiles were correlated with each other we visualized the emission rate per flower of each volatile in a heatmap and grouped volatiles by hierarchical clustering analysis of Pearson distances. The clustering analysis identifies sets of volatiles that have similar patterns among and within experimental groups, which could indicate shared biochemical regulation or genetic correlations. The heatmap is useful for assessing which volatiles are present in each group, as well as their emission rates and rarity.

### Moth attraction to floral scent

One way of testing whether a pollinator uses scent is to remove visual cues like color, size and shape by enclosing flowers in bags or other structures to hide them. We chose this general approach, enclosing flowers in mesh bags, similar to the approach of several previous studies in other systems (Knudsen et al., 1999; Raguso and Willis, 2002, 2005; Dötterl et al., 2012; Riffell and Alarcón, 2013; Barragán‐Fonseca et al., 2020). Such an approach isolates the role of scent, although it does not determine exactly which compounds are attractive, as can be done by enclosing synthetic scent blend emitters within mesh bags (e.g., El‐Sayed et al., 2008).

We used choice tests to assess whether the evening scent of *S. kaalae* attracts moths against a background of the scent and visual cues of one of two wind‐pollinated species (*S. kealiae* or *S. globosa*). These species produce strong floral scents reminiscent of butter and old socks (Jürgens et al., 2012, Powers et al., 2022, unpublished data) that are distinct from both *S. kaalae* and *S. hookeri*, and their inflorescences were previously shown to be relatively unattractive to moths in choice tests against *S. kaalae* (Weller et al., 2017). Choice tests experiments were performed at an experimental outplanting of *Schiedea kaalae* plants spaced c. 0.5 m from each other in an approximately 20 × 20 m plot in ‘Ēkahanui Gulch, Oʻahu, Hawaii, USA (outplanting described in Weisenberger et al., 2014 and Weller et al., 2017). Choice sticks were constructed as in Weller et al. (2017). The apparatus consisted of two florist tubes each containing either a *S. globosa* or *S. kealiae* inflorescence, with each tube taped to the ends of a 0.45 m horizontal stick. A perpendicular 1 m stick served as a handle. For each choice test we used two inflorescences of the same species and sex of approximately the same size. Inflorescences of both sexes of *Schiedea kealiae* (Weller & Sakai 791, Kealia Trail, cultivated) and *S. globosa* (Weller & Sakai 844, Makapu'u, natural) were used. These inflorescences were unbagged to provide a landing place for the moths. A portion of a *S. kaalae* inflorescence with 2 or 3 flowers (cut from plants at the experimental outplanting site) was inserted into the florist tube on one side of the choice stick and then enclosed in a white mesh organza bag (mesh size 0.37 mm, 27 holes per cm) to shield it from view. Organza bags allowed the passage of volatiles, based on observations of moths approaching bagged flowers in a previous experiment (Weller et al., 2017). A similar empty bag was placed on the opposite side as a control in case the white bag was attractive. For each side of the choice stick, we recorded *P. brevipalpis* approaches (flight within 10 cm) and visits (landings on the exposed inflorescence of *S. globosa* or *S. kealiae* or on the mesh bag). Visitation to this experiment likely reflects short‐distance (<10 m) attraction of moths already foraging in the patch, where they might already be attracted by the larger plume of *S. kaalae* floral odor. We performed 10 choice tests (16 observation hours) with *S globosa* and 18 choice tests (25 observation hours) with *S. kealiae* over 6 nights from 30 March to 4 April 2019. The air temperature was approximately 18°C with overcast or clear skies. Each choice test was observed for at least 30 min between 17:30 and 20:00 HAST, after the first moth was sighted within the outplanting site. We did not attempt to track individual moths. Moths were identified in the field by observers with prior experience with *P. brevipalpis*, and some were photographed for later verification by the authors and by Kyhl Austin on iNaturalist (GBIF.org,; 2025). To assess preference for *S. kaalae* scent, the proportions of approaches or visits were analyzed with generalized linear models using the R package glmmTMB (Brooks et al., 2017). We assumed a beta‐binomial distribution to account for overdispersion and used each choice test as the unit of replication, weighting by the total number of visits to that choice test. A generalized linear hypothesis test tested the null hypothesis that the mean is 50% using the R package *emmeans* (Lenth et al., 2018).

## RESULTS

### Floral scent of hybrids

**Figure 1:**
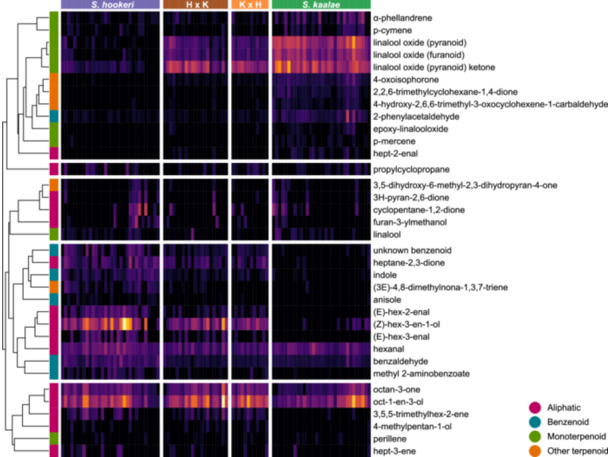
Patterns of floral volatiles in *Schiedea kaalae*, *S. hookeri*, and reciprocal hybrids. The emission rate per flower is shown by color (increasing from black to purple to yellow). Columns represent evening samples (1–4 samples per plant) and are ordered within the species or hybrid groups by scores along a one‐dimensional NMDS ordination. Rows correspond to individual volatile compounds (that occur in >13% of the samples), and volatiles are grouped by WPGMA hierarchical clustering analysis of Pearson distances. The class of each volatile is color‐coded at the left. The clustering reflects how volatiles are correlated within and across sampling groups and is cut at a fixed height into 5 blocks.

**Figure 2:**
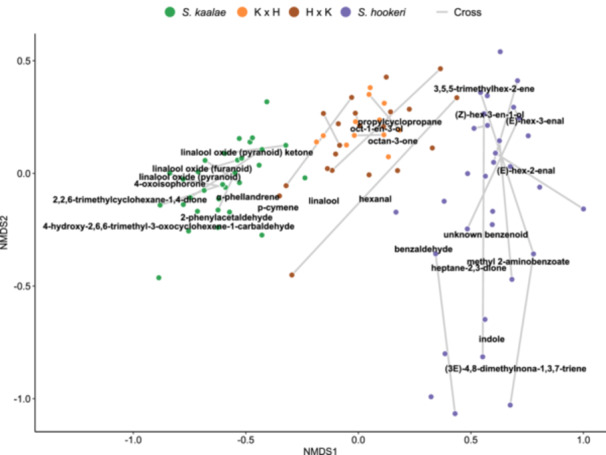
Differences in floral scent composition of evening samples of plants of *Schiedea kaalae*, *S. hookeri*, and reciprocal hybrids, visualized by NMDS of Bray‐Curtis dissimilarities between square‐root transformed relative emission rates (stress = 0.11). Samples are connected by lines if collected from different individuals of the same genotype (produced by vegetative propagation), or the same cross, defined by exact parentage. Resamples of the same individual are averaged prior to analysis (see Appendix S4 for within‐plant variation). Volatiles that occurred in more than 20% of samples are labeled at their weighted position in the ordination. For readability, the label for 4‐oxoisophorone has been shifted down and the label for propylcyclopropane has been shifted up.

**Figure 3:**
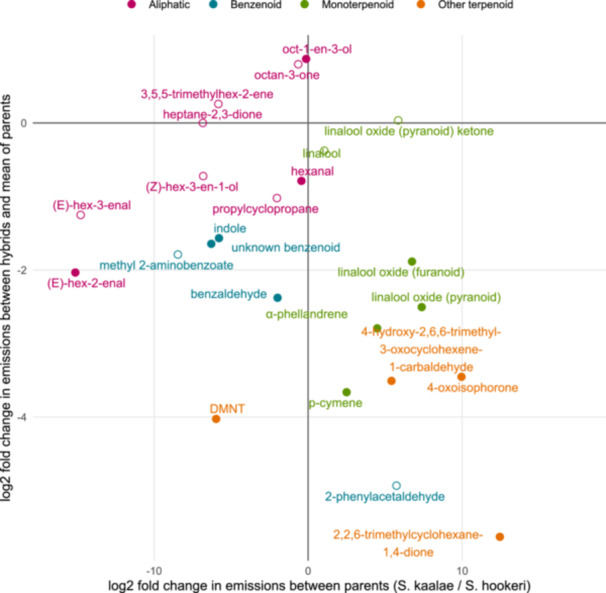
Volatiles arranged by how their emissions differ among hybrids and parent species. Axes are shown as the log_2_ fold change, where positive values on the y‐axis indicate higher emissions in hybrids than the mean of the two parent species and positive values on the x‐axis indicate higher emissions of *S. kaalae* than *S. hookeri*. Filled points indicate significant departures (P < 0.05) above or below an additive expectation that the hybrids produce each volatile at a level halfway between the levels of the parents (horizontal line). Volatiles are colored by compound class, with the “other terpenoid” category composed of sesquiterpene and carotenoid derivatives.

The two parent species produced qualitatively distinct scent blends in the evening (Figures 1, 2, 3; Powers et al., 2020), with the scent of *S. kaalae* dominated by three cyclic linalool oxide monoterpenoids (67% of the total blend) with relatively minor amounts of aliphatics and phenylacetaldehyde, and the scent of *S. hookeri* composed of oct‐1‐en‐3‐ol (41%) with a diverse set of aliphatics, benzenoids, and terpenoids as minor constituents. F_1_ hybrid plants produced a subset of volatiles drawn from the volatiles of both parent species (Figure 1), with all parental volatiles found in at least some hybrids. No novel hybrid volatiles were detected in two or more samples. As examples, most hybrids produced the volatiles benzaldehyde, hexanal, octan‐3‐one, and oct‐1‐en‐3‐ol produced by both species, the three linalool oxides and 4‐oxoisophorone produced primarily by *S. kaalae*, and indole, the unknown benzenoid, and heptane‐2,3‐dione produced primarily by *S. hookeri* (Figure 1; Appendix S5).

**Figure 4:**
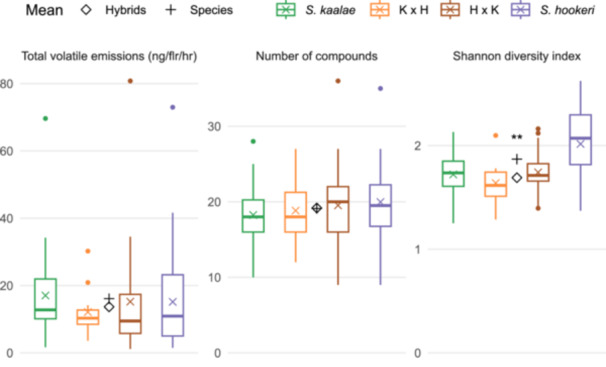
Total volatile emissions rates per flower, number of compounds, and Shannon diversity index for *Schiedea kaalae*, *S. hookeri*, and reciprocal hybrids (denoted by maternal then paternal parent). Boxplots show quartiles and medians, with means shown by an X. The mean of the two parent species means is indicated by a plus sign, and the mean of the two reciprocal hybrids means is indicated with a diamond, with a statistically significant difference between them indicated by the number of asterisks (**P < 0.01). Total volatile emissions per inflorescence are higher, i.e., the species (interpopulation crosses) and hybrids produce the following numbers of flowers per inflorescence in the greenhouse (median and interquartile range, n = 859 plants): *S. kaalae* 332 (184–504), *S. hookeri* 26 (19–34), K × H hybrids 126 (67–237), H × K hybrids 201 (98–336).

Hybrids did not differ significantly from the additive expectation (the mean of the parents) in their total volatile emissions (P = 0.44) or number of compounds (P = 0.95), but the mean Shannon diversity in hybrids (1.7) was slightly lower than the mean of the parents (1.9, P = 0.002, Figure 4). For the 24 compounds found in at least 25% of the samples, the emission rates in hybrids were either significantly above (1 compound), below (13 compounds), or indistinguishable from the additive expectation (10 compounds with P > 0.05 with the false discovery rate method; Figure 3; Appendix S5). The four compounds that were emitted at similar levels in both parents had contrasting fates in hybrids, i.e., compared to the parent mean, hybrids emitted 83% more of mushroom‐scented 1‐octen‐3‐ol but 42% less hexanal, with 3‐octanone and linalool produced at similar mean amounts as the parents (Figure 3). Six of the nine aliphatic compounds were produced at levels indistinguishable from the parent mean, and most were produced at higher amounts in *S. hookeri* than in *S. kaalae*. Hybrids produced benzenoids, monoterpenoids, and other terpenoids at levels generally below the additive expectation. In many of these cases, the compound was produced predominantly by one parent (Figure 3). For example, the carotenoid derivative 2,2,6‐trimethylcyclohexane‐1,4‐dione was common in *S. kaalae* and the homoterpene (3E)−4,8‐dimethyl‐1,3,7‐nonatriene (DMNT) was common in *S. hookeri*, but each was rarely produced by hybrids (Appendix S5; Figure 1).

The two hybrid cross directions (the species of the maternal parent) did not differ in their total volatile emissions (P = 0.56) Shannon diversity (P = 0.26), or number of compounds (P = 0.70, Figure 4). The cross directions did not detectably differ in emissions of any particular compound (all P > 0.28, Appendix S5). There was no detected separation of relative scent composition by hybrid cross direction (PERMANOVA P = 0.98, Figure 2).

In a multivariate analysis of relative volatile emissions (fractions of the total emissions), most hybrids produced intermediate scent compositions relative to those of the two species, although a few hybrids produced scent compositions that overlap with the range of parents’ scents, based on the NMDS analysis (Figure 2). The hybrid plants showed a magnitude of interplant variation similar to *S. kaalae*, and lower than the interplant diversity of *S. hookeri* (analysis of multivariate homogeneity of group dispersions P < 0.001; Appendix S6; Figure 2). Different inflorescences from the same individual (averaged in other analyses) yielded similar scent compositions (Appendices [Link], [Link]).

Some volatiles were highly correlated with each other across plants (Figure 1), which could indicate they are under shared regulation, are genetically correlated, or merely co‐occur in one species or the other. For example, all the monoterpenes (including the linalool oxides) and 2‐phenylacetaldehyde cluster together (Figure 1, block at the top), which could be due to common regulation or the fact that they occur together in *S. kaalae* and hybrids but are largely absent from the scent of *S. hookeri*. Most of the volatiles in the fourth block of volatiles occur in *S. hookeri* and hybrids but not *S. kaalae*, and these compounds originate from diverse biochemical pathways (green‐leaf aliphatics, benzenoids, and nitrogenous compounds; Dudareva et al., 2004). The other blocks contain compounds with similar emissions in both parents and hybrids.

### Moth attraction to floral scent

**Figure 5:**
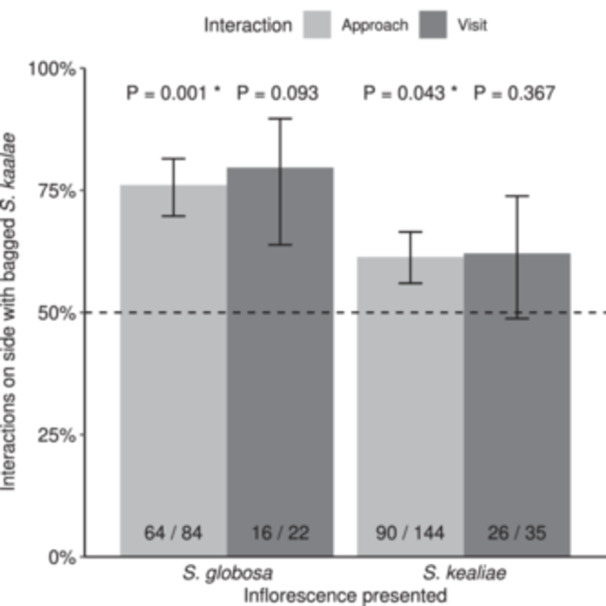
Preferences of the moth *Pseudoschrankia brevipalpis* for wind‐pollinated *Schiedea* inflorescences (either *S. globosa* or *S. kealiae*) augmented with bagged *S. kaalae* versus controls with empty bags. P‐values test the null hypothesis of no preference (μ = 50%, dashed line) according to a beta‐binomial generalized linear model. Bars indicate the estimated marginal means and standard errors of the proportion of approaches (light grey) or visits (dark grey). The number of interactions on the side with bagged *S. kaalae* is expressed as a fraction of the total at the bottom of the bar (raw fractions differ from the modeled estimated marginal means). With *S. globosa*, there were 10 choice tests over 16 observation hours, and for *S. kealiae* there were 18 choice tests over 15 observation hours.

We tested the attractive ability of the scent of *Schiedea kaalae* against empty bag controls, in a total of 28 choice tests that spanned 40 observation hours. Across the entire experiment, moths only approached and visited 57 times compared to approaching without visiting 228 times (14 to 23% of approaches led to visits, depending on the combination of species). Moths preferred to approach and visit *S. globosa* inflorescences augmented with *S. kaalae* scent compared those with empty bag controls (77% of approaches and 73% of visits were to the *S. kaalae* side of the choice stick, Figure 5). Moths also preferred to approach (59%) and visit (74%) *S. kealiae* inflorescences augmented with *S. kaalae* scent compared to those with empty bag controls (Figure 5). In both cases this preference was statistically significant for approaches but not visits (Figure 5).

## DISCUSSION

### Hybrid scent is intermediate between parents

As a group, hybrids of sympatric *Schiedea kaalae* and *S. hookeri* produce nearly all the compounds that are shared by or unique to each parental species, at similar total levels and numbers of compounds per plant, without producing novel volatiles. Individual hybrid plants vary in which volatiles they produce, but are mostly intermediate in scent composition, with just a few individuals resembling either parent species. These results suggest that simultaneous production of the volatiles of both species is biochemically and genetically possible because the synthetic and regulatory genes from both parents are active in hybrids. In these hybrids, expression of these genes from a single chromosome set is sufficient to allow at least some scent production and is not totally inhibited by genes on the chromosome of the other parent.

This qualitative pattern of complementary expression, where hybrids produce compounds from both parents, is well known and common in leaf secondary metabolites (reviewed in López‐Caamal and Tovar‐Sánchez, 2014), but less well characterized for floral volatiles. Several studies have measured floral scent in both parent species and F_1_ hybrids of known parentage, in the context of breeding ornamental flowers (Fu et al., 2007; Wei et al., 2021) and in nature (Hirota et al., 2012; Campbell et al., 2022; García et al., 2023). However, the low sample sizes and focus on total scent intensity in some of these studies (Hirota et al., 2012; Wei et al., 2021) preclude rigorous comparisons that could shed light on volatile inheritance patterns.

In this study, we examined a large sample of F_1_ hybrids (N = 33) to examine how volatile compounds varied in their quantitative patterns of inheritance. Just under half of the compounds conformed to the expectation of additive genetic variation (had emissions that were halfway between the parents), and just over half fell below this expectation, consistent with recessive inheritance of the scent allele. One compound exceeded the additive expectation, with mean emissions above those of either parent, consistent with transgressive expression. This distribution of inheritance patterns aligns well with those broadly observed for leaf secondary chemistry, where most metabolites are expressed at levels similar to or intermediate to the parent species, and only rarely fall outside the range of the parents (López‐Caamal and Tovar‐Sánchez, 2014).

In the hybrids, neither total scent emissions nor scent composition depended on the direction of the cross, indicating that reciprocal effects were absent. Hybrids germinating in populations of either species would have similar floral scents. Thus far, only nuclear genes have been reported to affect scent production (Borghi et al., 2017), which is consistent with this result.

Scent reduction or novelty that makes hybrids unrecognizable or attractive to a new species of pollinator have been identified as postzygotic reproductive barriers (Marques et al., 2016), but those two possibilities can be ruled out in hybrids of this *Schiedea* species pair. The implications of intermediate scent for reproductive isolation are still unclear because the attractive ability of each individual volatile or suite of volatiles has not yet been established. If moths (*Pseudoschrankia brevipalpis*) can respond to the volatile blend of each species regardless of competing signals from the other species, then hybrids that produce a mixture of the two species’ scents should still be attractive to moths. The choice test experiments with bagged *S. kaalae* suggest that moths may have this ability since they were attracted to *S. kaalae* scent when mixed with scent from a wind‐pollinated species (both wind‐pollinated species have scents distinct from *S. kaalae*; Jürgens et al., 2012; Powers et al., 2020, 2022). Intermediate scent blends (and blends occasionally overlapping with the scent of a parent species) were also observed in natural hybrids of two species of Joshua tree (*Yucca*) at a secondary contact zone, and genetic evidence of backcrossing indicates that moths must have visited those hybrids (Svensson et al., 2016). In the case of two orchids that attract the same bee pollinator by sexual deception, hybrids also attracted that pollinator by producing the same set of antennally active compounds as the two parent species, and an intermediate blend of the non‐active compounds (Cortis et al., 2009). Alternatively, effects of intermediate blends may be unexpected, i.e., hybrids of two bee‐pollinated sexually deceptive orchids produced a combination of parental volatiles (and two new compounds out of 73), which made them attractive to a new butterfly pollinator (Vereecken et al., 2010). Ultimately, choice tests of *Schiedea* hybrids versus parents are required to confirm that hybrids are not reproductively isolated from their parents by their intermediate scent blend (Ma et al., 2016).

### Moths attracted to floral scent

We established experimentally that the scent of *Schiedea kaalae* is attractive to the endemic Hawaiian moth *Pseudoschrankia brevipalpis*, as has been demonstrated for other moth pollinators using floral scent or artificial scent mixtures with visual cues blocked (Dötterl et al., 2012; El‐Sayed et al., 2008). Because the flowers were bagged, specific visual signals (the appearance of *S. kaalae* inflorescences or flowers) are probably not required for approaches or visitation in this moth species. However, stronger tests of this hypothesis would require application of artificial or extracted scents from *S. kaalae*. A small number of *S. kaalae* flowers were able to attract moths against a background of competing scent and visual presentation from wind‐pollinated inflorescences that have been previously shown to be relatively unattractive to these moths (Weller et al., 2017). These results indicate that moths can use the species‐specific scent of *S. kaalae* to locate flowers in this food‐rewarding pollination system. Most volatiles that *S. kaalae* produces in the evening are typical of moth‐pollinated taxa in other plant families (Powers et al., 2020), so it is likely that they play a role in long‐ or short‐distance attraction.

Moths visited (landed on the bag or wind‐pollinated inflorescence) only 14 to 23% of the time that they approached a side of the choice stick. It is plausible that their landing behavior is suppressed by the unfamiliar or unattractive visual cues of the wind‐pollinated inflorescences, or the discordance between these visual cues and the scent cues of *S. kaalae*. Discrimination was incomplete, with moths sometimes approaching (23 to 41% of the time) or visiting (26 to 27%) the inflorescences without added *S. kaalae* flowers. The incomplete preference could be due to the diffuse *S. kaalae* scent present in the population, or any vestigial attractive ability of the wind‐pollinated species inflorescences (visual or olfactory). During transitions to wind pollination within genera, wind‐pollinated taxa are known to lose volatiles that provoke antennal responses in pollinators (Wang et al., 2019).

Experiments that test the attractive ability of single volatiles or sets of volatiles could identify the components of the scent blend necessary for moth attraction, and if successful provide a rapid bioassay for assessing pollinator presence at potential restoration sites. These tests would also reveal whether moths are attracted to volatiles shared across moth‐pollinated *Schiedea*, or if they use species‐specific compounds. A study of *Cirsium* (Asteraceae) volatile mixes showed that while one compound (phenylacetaldehyde, which increases in *S. kaalae* in the evening), was sufficient to attract small moths, the addition of minor compounds increased visitation (El‐Sayed et al., 2008). It follows that both single general attractants and other reinforcing volatiles may be important for moth attraction. Finally, the preference for *S. kaalae* over *S. hookeri* at a site planted with *S. kaalae* suggests the hypothesis that scent or visual cues are learned, i.e., preferences are produced or reinforced by associating specific floral traits with a reward. Visual traits may still play a minor role, i.e., while hawkmoths learn to associate nectar more strongly with olfactory cues than visual ones, the presence of both reduces decision times (Riffell and Alarcón, 2013).

### Contrasting hybrid zones

While we studied a case where reproductive barriers have evidently stopped the formation or persistence of hybrids in nature, a comparative study of other *Schiedea*, some of which form hybrid zones (Weller et al., 2001), may yield different patterns of parental versus hybrid scent. For example, gynodioecious, wind‐pollinated *S. salicaria* Hillebr. has produced a hybrid swarm with hermaphroditic *S. menziesii* Hook. which is presumably biotically pollinated but currently selfing (Wallace et al., 2011). Due to asymmetric gene flow of pollen of *S. salicaria* to *S. menziesii*, natural hybrids have a nuclear genome, morphology, and sex expression similar to *S. salicaria*, but the chloroplast genome of *S. menziesii* (Wallace et al., 2011). We predict that hybrids would also have a floral scent similar to wind‐pollinated *S. salicaria* due to replacement of the nuclear genome of *S. menziesii*, and if so, be less attractive to any biotic pollinators. This would intensify selection for wind pollination in the hybrid zone. In other cases, particularly on older islands, the greater genetic distance between species may cause hybrids to have genetic incompatibilities that result in reduced floral scent and represent a postzygotic reproductive barrier, if the species are biotically pollinated.

## CONCLUSIONS

Controlled crosses showed that F_1_ hybrids between two moth‐pollinated species of *Schiedea* produced scents that are largely intermediate between those of the two parental species, or similar to one parental species suggesting dominance effects, with no effect of the direction of the cross. The moth pollinator uses scent to find inflorescences, and the non‐transgressive scent suggests they would potentially be attracted to F_1_ hybrids as well as the parent species, showing the importance of floral scent variation in a system dependent upon pollination by small moths.

## AUTHOR CONTRIBUTIONS

All authors participated in the design of the experiments. D.R.C provided advice on scent collection and analysis. J.M.P, S.G.W., and D.R.C. conducted the choice tests. J.M.P. collected and analyzed the scent data and wrote the manuscript. All authors contributed substantially to revisions.

## Supporting information


**Appendix S1.** Photos of flowers.

**Appendix S2.** Sampling localities.

**Appendix S3.** Crossing design.

**Appendix S4.** Ordination of within‐plant variation in floral scent.

**Appendix S5.** Emission rates of all compounds.

**Appendix S6.** Levels of among‐plant variation.

## Data Availability

Data and code used for generating figures and conducting analyses are available at: https://github.com/jmpowers/schiedea‐scent.
