# Supplementary material for: Floral scent of artificial hybrids between two Schiedea species that share a moth pollinator
Source: Am J Bot. 2025 Jun 29;112(7):e70065. doi: 10.1002/ajb2.70065 (PMC12281260; doi:10.1002/ajb2.70065)

Appendix S1. Photos of flowers

Male-phase flowers of *Schiedea kaalae*, *S. hookeri*, and a F1 hybrid, showing the sepals, tubular nectary extensions (white arrows), stamens, and pistils. The sepals of *S. kaalae* and *S. hookeri* are 3-4 mm long (Wagner et al. 2005).

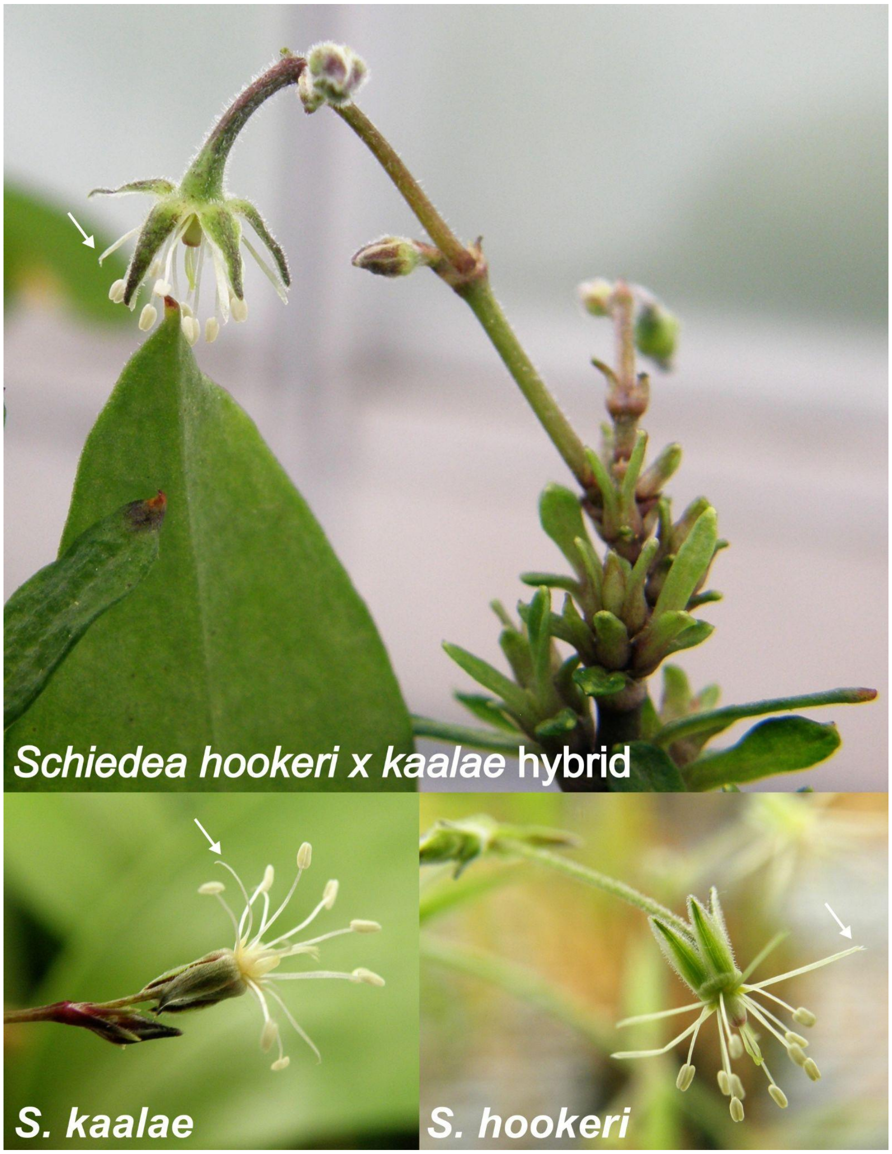

Supplement: Supplementary file 1 — Appendix S1. Photos of flowers. [file AJB2-112-e70065-s003.pdf]
