# Supplementary material for: Floral scent of artificial hybrids between two Schiedea species that share a moth pollinator
Source: Am J Bot. 2025 Jun 29;112(7):e70065. doi: 10.1002/ajb2.70065 (PMC12281260; doi:10.1002/ajb2.70065)
Supplement: Supplementary file 2 — Appendix S2. Sampling localities. [file AJB2-112-e70065-s002.pdf]

Appendix S2. Sampling localities

Localities of *Schiedea* populations in this study (Wagner *et al.* 2005). Collections of *S. hookeri* from Wai‘anae Kai were treated as a single population (WK) for this study.

**Species**	**Range**	**Location**	**Population collection number**
S. hookeri	Wai‘anae	Kalua‘a Gulch, S of Pu‘uhapapa	Weller and Sakai 879 (BISH, US)
		Wai‘anae Kai, various locations	WK (Weller and Sakai 794, BISH;<br>Weller and Sakai 866, US;<br>Weller and Sakai 891, BISH PTBG, US;<br>Weller and Sakai 899, US)
S. kaalae	Wai‘anae	Kalua‘a Gulch, S of Pu‘uhapapa	Weller and Sakai 892 (US)
		Pahole Gulch	Weller and Sakai 904 (BISH, PTBG, US)
		E of Pu‘ukaua, near Pu‘umaialau	Takeuchi 3587 (BISH)
	Ko‘olau Range	Makaua Valley (Hidden Valley)	Weller and Sakai 881 (BISH, PTBG, US)

Reference

Wagner, W. L., Weller, S. G., and Sakai, A. (2005). Monograph of *Schiedea* (Caryophyllaceae subfam. Alsinoideae). *Systematic Botany Monographs* 72, 1–169.
