# Supplementary material for: Floral scent of artificial hybrids between two Schiedea species that share a moth pollinator
Source: Am J Bot. 2025 Jun 29;112(7):e70065. doi: 10.1002/ajb2.70065 (PMC12281260; doi:10.1002/ajb2.70065)
Supplement: Supplementary file 3 — Appendix S3. Crossing design. [file AJB2-112-e70065-s004.pdf]

Appendix S3. Crossing design

Schiedea plants sampled for floral volatiles (n = 32 *S. hookeri*, 12 *S. kaalae* x *S. hookeri*, 21 *S. hookeri* x *S. kaalae*, 32 *S. kaalae*), organized by their maternal and paternal parents. The number of maternal (n = 19) or paternal plants (n = 22) used to make the crosses are given in parentheses next to the population number. On the diagonal, the number of plants from within-population crosses is given first and the number of sampled plants that originated from the field (as seeds or cuttings) is given second. Shading indicated the cross type. All populations occur in the Waiʻanae Mts. except 881 which occurs in the Koʻolau Mts (see Appendix S2 for population localities).

Maternal		Paternal					
		S. hookeri		***S. kaalae***			
		879 (4)	WK (8)	3587 (4)	892 (3)	904 (3)	881 (0)
S. hookeri	879 (4)	10 + 1	2	3	1	3	
	WK (4)	4	6 + 9	4	6	5	
S. kaalae	3587 (5)	2	3	5 + 4		3	
	892 (3)			3	3 + 1	1	
	904 (3)	4	2	2	1	4 + 2	
	881 (0)						0 + 3
