# Supplementary material for: Floral scent of artificial hybrids between two Schiedea species that share a moth pollinator
Source: Am J Bot. 2025 Jun 29;112(7):e70065. doi: 10.1002/ajb2.70065 (PMC12281260; doi:10.1002/ajb2.70065)

Appendix S4. Ordination of within-plant variation in floral scent

Ordination of evening scent samples of *Schiedea kaalae*, *S. hookeri*, and reciprocal hybrids with resamples of plants shown. The NMDS uses Bray-Curtis dissimilarities between square-root transformed relative emission rates (stress = 0.11). Samples are connected by lines if collected from the same sampling bag at the same time, the same inflorescence on different dates, the same plant on different dates, different individuals of the same genotype (produced by vegetative propagation), or the same cross, defined by exact parentage. Volatiles that occurred in more than 20% of samples are labeled at their weighted position in the ordination.

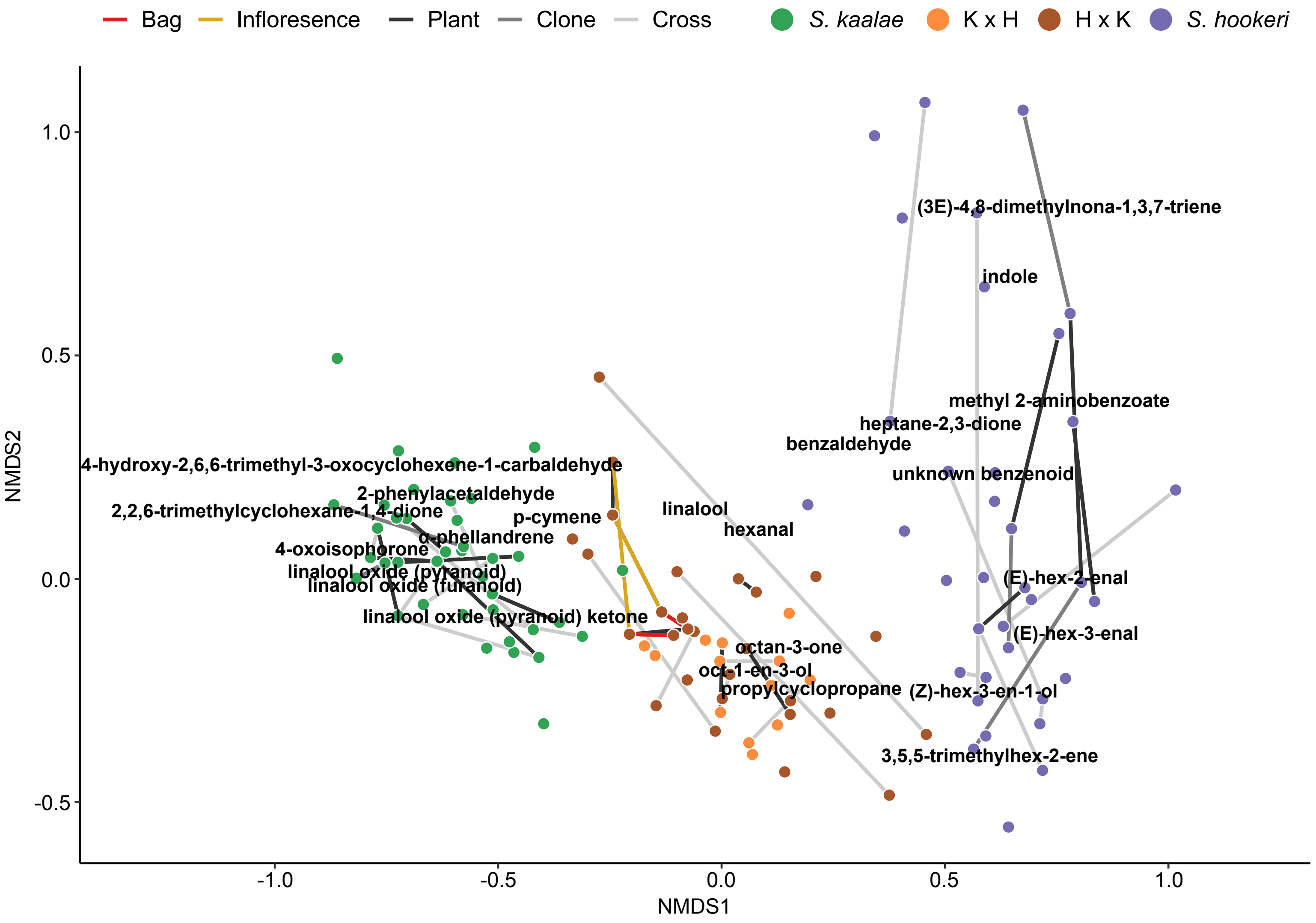

Supplement: Supplementary file 4 — Appendix S4. Ordination of within‐plant variation in floral scent. [file AJB2-112-e70065-s001.pdf]
