# Supplementary material for: Floral scent of artificial hybrids between two Schiedea species that share a moth pollinator
Source: Am J Bot. 2025 Jun 29;112(7):e70065. doi: 10.1002/ajb2.70065 (PMC12281260; doi:10.1002/ajb2.70065)

Appendix S5. Emission rates of all compounds

Volatile emission rates per flower (square root scale) for *Schiedea kaalae*, *S. hookeri*, and reciprocal hybrids (denoted by maternal then paternal parent). Boxplots show quartiles and medians, with means shown by an X. The mean of the two parent species means is indicated by a plus sign, and the mean of the two reciprocal hybrids means is indicated with a diamond, with a statistically significant difference between them indicated by the number of asterisks (* P < 0.05, ** P < 0.01, *** P < 0.001). Volatiles occurring in > 20% of samples are arranged (left to right and top to bottom) by an index of how the mean emissions of hybrids relates to the means of the two parent species (above, between, below).

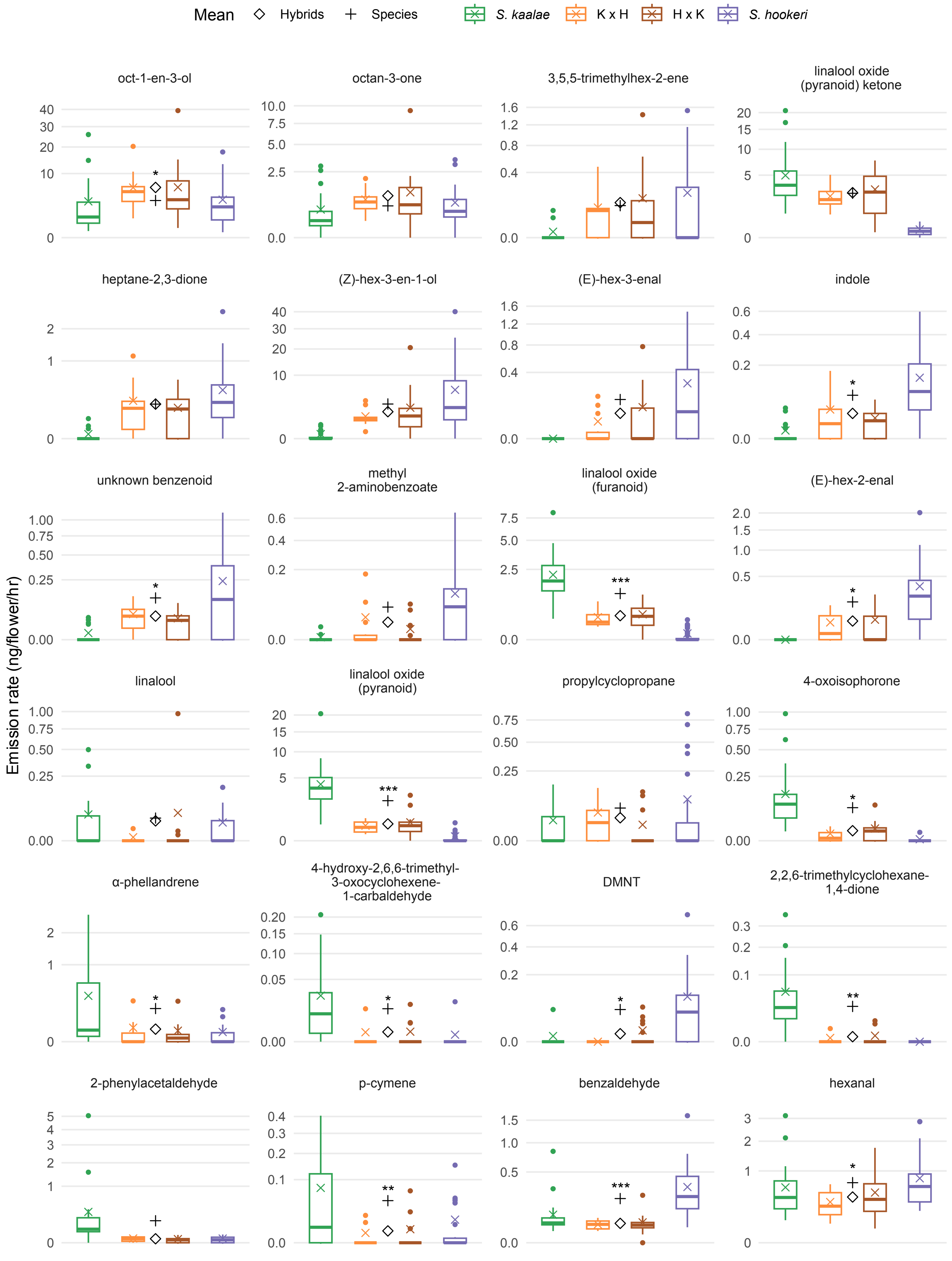

Supplement: Supplementary file 5 — Appendix S5. Emission rates of all compounds. [file AJB2-112-e70065-s006.pdf]
