# Supplementary material for: Floral scent of artificial hybrids between two Schiedea species that share a moth pollinator
Source: Am J Bot. 2025 Jun 29;112(7):e70065. doi: 10.1002/ajb2.70065 (PMC12281260; doi:10.1002/ajb2.70065)

Appendix S6. Levels of among-plant variation

Levels of among-plant variation in relative floral scent composition for hybrids and parent species. Points indicate the Bray-Curtis distance from each plant to the centroid of the group (with emissions of multiple inflorescences averaged). The level of within-plant variation is given by the distances of multiple inflorescences to the centroid of each plant that was resampled (29 inflorescences from 13 plants). Boxplots show medians and quartiles.

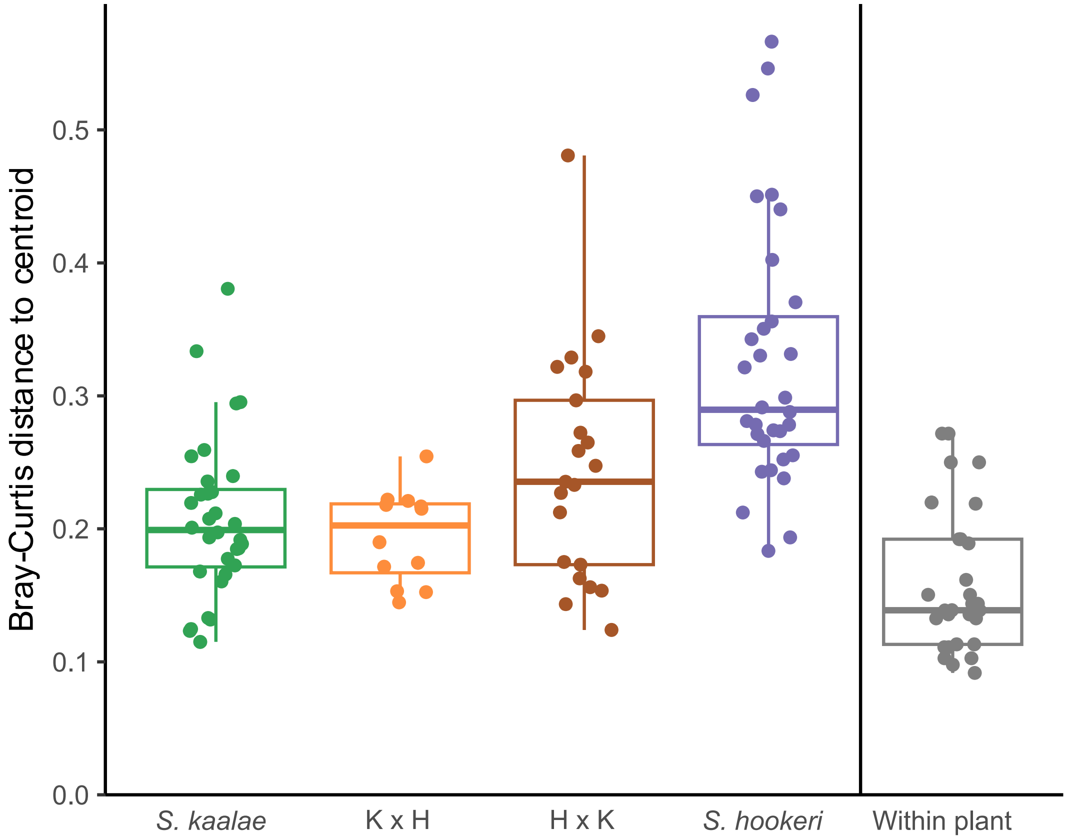

Supplement: Supplementary file 6 — Appendix S6. Levels of among‐plant variation. [file AJB2-112-e70065-s005.pdf]
